# Prevalence of Transient Hypothyroidism in Children Diagnosed with Congenital Hypothyroidism between 2000 and 2016

**DOI:** 10.3390/ijms24032817

**Published:** 2023-02-01

**Authors:** Sabrina Gmür, Daniel Konrad, Ralph Fingerhut

**Affiliations:** 1 Department of Endocrinology & Diabetology, University Children’s Hospital Zurich, University of Zurich, CH-8032 Zurich, Switzerland; 2 Children’s Research Center, University Children’s Hospital Zurich, University of Zurich, CH-8032 Zurich, Switzerland; 3 Swiss Newborn Screening Laboratory, University Children’s Hospital, CH-8032 Zurich, Switzerland

**Keywords:** congenital hypothyroidism (CH), newborn screening (NBS), prematurity, total thyroxine (TT4), thyroid stimulating hormone (TSH), dried blood sample (DBS)

## Abstract

Newborn screening (NBS) for congenital hypothyroidism (CH) was introduced in Switzerland in 1977, which allowed for the preclinical, biochemical diagnosis. The aim of this study was to evaluate the prevalence of transient CH (tCH) in the canton of Zurich. In this analytical cohort study, all newborns born in the canton of Zurich, between the 1st of January 2000 and the 30st of June 2016, with a TSH value above 15 mU/L (whole blood) were included. There were 115 cases out of 247,918 babies born during the study period. However, 23 cases had to be excluded due to missing data. The definite diagnosis was made after a thyroxine withdrawal at 2 years of age. The total prevalence of confirmed CH and the female to male ratio (f/m) were 1:2695 and 2.17:1; for permanent CH (pCH), 1:3443 and 2.8:1; and for tCH, 1:12,396 and 1:1, respectively. The TSH value was significantly higher in pCH compared to tCH, at 130.3 (62.9–171.9) and 36.4 (26.5–53.3) (median and interquartile range), respectively (*p* < 0.001). The prevalences found for congenital hypothyroidism and its transient form are comparable to previous studies. TSH concentration at birth was predictive for the further course of the disease. Low birth weight correlated with a tCH, whereas low gestational age did not. The dominance of the female sex in congenital hypothyroidism is supported by a gender ratio of 2.17:1.

## 1. Introduction

Congenital hypothyroidism (CH) is the most prevalent reason for mental retardation, if untreated, and the most frequent endocrinopathy, with an incidence of 1:3000–1:4000 newborns [1,2,3,4,5,6,7,8,9,10]. Clinical signs are respiratory insufficiency, cyanosis, icterus praecox umbilicalis, cretinism, muscular hypotonia, a wide fontanel, and psychomotor retardation. Most newborns with CH show no clinical signs at birth, since many have residual thyroid function, and, in addition, the partial transplacental passage of maternal thyroid hormones offers temporary protection [8]. Normal brain development in utero as well as postnatally is mainly dependent on an adequate supply of thyroid hormones [7,11]. Already during the first trimester, thyroid hormone receptors are expressed in the embryonic brain [11]. The first symptoms normally do not appear before the 4th to 12th week of life, however, parents sometimes report a higher sleepiness and feeding problems. If the patients remain untreated until adulthood, they develop dwarfism with a short stature and short extremities, delayed speech development, and reduced IQ with profound mental retardation. The majority of patients with CH need lifelong substitution with tyroxine, which is referred to as permanent CH (pCH), however, in a significant number of patients, thyroid function recovers in early childhood. These patients are the so-called transient CH (tCH) [12].

The introduction of NBS for CH has dramatically changed the situation, since NBS provides a rapid diagnosis, and early treatment with hormone replacement therapy provides a normal development. Only 5% of newborns with CH are already clinically suspicious before the screening result is available [3]. NBS for CH has a very good cost-benefit ratio [4,5,6,7].

Studies from 2007 through 2010 suggested that the prevalence of CH is increasing [13,14]. This apparent increase could be due to several reasons, for example, decreased TSH cut-offs, increasing maternal age, higher number of prematurity, and multiple pregnancies [7,13,14,15,16,17]. CH also varies in different ethnicities [7], however it is unclear whether this variation only affects pCH or also tCH [18].

Transient CH can have several causes, with iodine probably being one of the most important. Iodine containing disinfectants, or contrast medium, applied in the neonatal period, and an iodine rich diet or medication can cause iodine overload in the newborn. Additionally, maternal exposure to iodine can, either due to placental transfer or via breast milk, result in high iodine in the newborn. Additionally, iodine deficiency can lead to tCH, and in the worst case, when T4 is absent during early fetal life, to irreversible brain damage [19,20].

Previous studies have postulated that the prevalence of tCH lies between 5% and 65% of all diagnosed cases of CH [10,21,22,23,24,25]. Low birth weight (<1500 g), non-caucasian ethnicity, complications in the perinatal period, and less severe forms of CH have been discussed as possible reasons for this variability [18,22,23,24]. Primary CH may be due to a defect in hormonogenesis, dysgenesis, or ectopia of the thyroid, or athyreosis. Other forms are central CH, sometimes also called secondary and tertiary CH. These include defects in the secretion of TSH or thyrotropin releasing hormone (TRH). The prevalence of central CH is estimated to be 1:20,000–1:30,000 live births [26]. Central CH is not detected by NBS programs that measure TSH in dried blood samples (DBS). In contrast to all other disorders that are part of NBS programs, in the past, approximately only 2% of CH cases were ascribable to genetic defects [14,27]. However, in the meantime, the number of cases with a proven genetic cause has risen to approximately 20% [28,29,30].

NBS for CH was introduced in Switzerland in 1977. Until 2004 there were two NBS laboratories, one in Zurich at the University Children’s Hospital and a second in Berne at the blood transfusion service of the Swiss Red Cross. Since 2005, there has been one NBS laboratory in Switzerland at the university children’s hospital Zurich. In a retrospective study with children born between 1 January 2000 and 30 July 2016, in the canton of Zurich, we wanted to determine the prevalence of tCH in order to estimate whether the recently often postulated increase in the prevalence of CH might be due to the increased detection of tCH. In addition, we wanted to analyze whether TSH values are significantly higher in children with pCH, and whether there is any correlation of sex, gestational age (GA), and low birth weight with tCH.

## 2. Results

### 2.1. Study Design

For this analytical retrospective cohort study, patient data were extracted from the Swiss NBS laboratory and from the medical files of the University Children’s Hospital Zurich. Whole blood TSH and total T4 (TT4) values are from the NBS laboratory, and serum TSH and free T4 (fT4) values at 2 years of age are from the hospital medical file system (CompuGroup Medical Schweiz AG, Bern, Switzerland) and from microfilms archived in the University Children’s Hospital Zurich. In addition, further data were extracted from the medical files: sex; birth weight; gestational age; mode of delivery; family history concerning thyroid disease of first grade relatives; autoantibodies, especially TPO antibodies and TSH receptor antibodies (TSHR AB) of the newborn and the mother; and sonography examining the localization and morphology of the thyroid gland. The total number of live births in the canton of Zurich was taken from the federal bureau of statistics (Bundesamt für Statistik) in Bern.

The rational for this extensive retrieval of data is the fact that most studies only focus on selected topics or data. Our study, however, presents all the available data of the study cohort, and therefore we are able to prove or disprove the influence of the selected data, or at least discuss controversial findings.

### 2.2. Patient Characteristics

**Figure 1:**
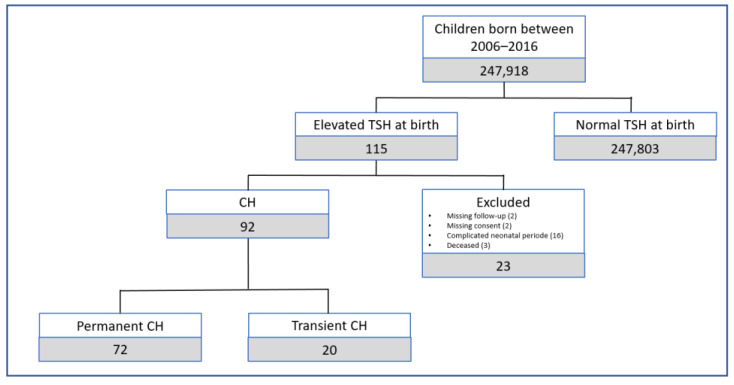
Number of patients included and excluded from the study.

According to the Swiss federal office for statistics, 247,918 babies were born between 2000 and 2016 in the canton of Zurich [31]. Between the 1st of January 2000 and the 30th of June 2016, 115 newborns revealed an elevated TSH concentration in the NBS sample. Of these 115 screen positive cases, 23 were excluded: two due to missing parental consent for the research; three children died within the first 2 years of life; and 16 children had a complicated neonatal period or were premature and had normal TSH values at follow-up. Therefore, a total of 92 children were included in our study (Figure 1).

**Figure 2:**
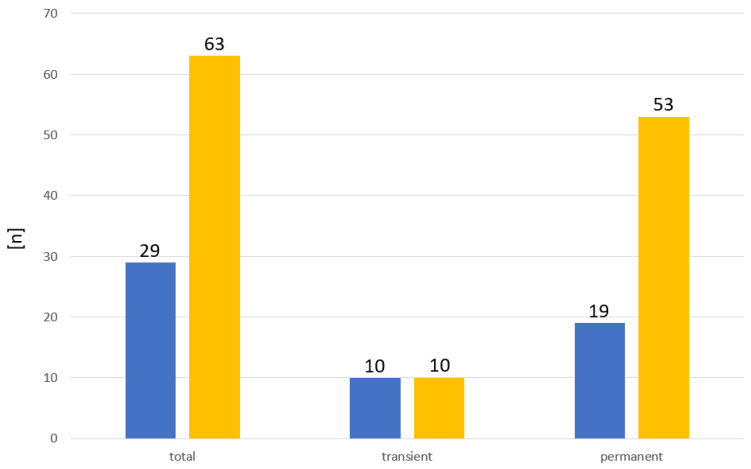
Sex distribution in patients with CH. ■ Male. ■ Female.

Of these 92 children that were included in our study, 29 were male (32%) and 63 were female (68%). In total, 72 (78%) had pCH and 20 (22%) had tCH (Table 1). The sex ratio for tCH was 1/1 (m/f), while it was 1/2.8 (m/f) for pCH (Figure 2). Due to the relative high number of cases excluded, the sex ratios might be biased. However, the number of male and female cases within the excluded cases was nearly equal.

At the University Children’s Hospital in Zurich, we normally perform a thyroxine withdrawal in all patients with thyroid tissue present in loco classico at the age of two years. This procedure is in accordance with current guidelines, which foresee that the reevaluation of the HPT axis is indicated after the age of 2 to 3 years when no definitive diagnosis of pCH has been made, particularly in children with gland in situ and with presumed isolated central CH [32]. However, in patients with low thyroxine requirements, a continuous reassessment at a later age may be carried out.

**Figure 3:**
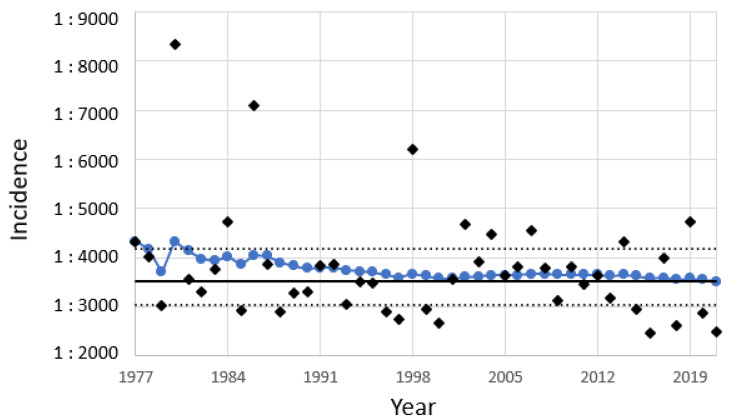
Incidence of CH in Switzerland from 1977 to 2021. ◆ Incidence per year; ● cumulative incidence from 1977 to the respective year; ― total incidence from 1977 to 2021; ^….^ 95% CI of the total incidence.

The total incidence of CH in Zurich was 1:2695 (1:2197–1:3343; 95% CI); for pCH it was 1:3443 (1:2734–1:4400; 95% CI); and for tCH it was 1:12,396 (1:8026–1:20,295; 95% CI). The total incidence of CH is slightly higher in Zurich than in the rest of Switzerland. The total incidence of CH in Switzerland has been permanently around 1:3513 (1:3028–1:4180; 95% CI, from 1977–2019) [Table 2, Figure 3]. Statistical analysis revealed no trend of the total incidence (*p* = 0.309, 2-sided linear regression; no trend, Von Neumann trend test).

### 2.3. Thyroid Autoantibodies

In 9 of the 20 cases with tCH (no. 1, 3, 4, 8, 11–15), thyroid autoantibodies could be detected in both the child and the mother. Therefore, a placental transfer of maternal autoantibodies to the fetus can be assumed in these nine cases (Table 3). In one case (no. 9) autoantibodies were detected in the mother, but the documentation was missing for the child. In two cases (no. 5, 10) there were no autoantibodies detected, neither in the child nor in the mother. In the remaining eight cases (no. 2, 6, 7, 16–20) there was no documentation on whether or not thyroid autoantibodies were measured. In 2 of the 20 cases (no. 3, 8), TSHR Ab are probably the reason for tCH, and one child had a suspicion of Noonan syndrome or a differential diagnosis of iodine induced tCH.

In some of the cases with pCH, it was not documented whether or not the child and/or the mother were tested for thyroid autoantibodies. However, in 10 of the 72 cases with pCH, autoantibodies could be detected (Table 4). In 6 of these 10 cases, there was no thyroid tissue detected sonographically. In the other four cases, sonography of the thyroid was normal. In 34 cases no thyroid autoantibodies were detected, neither in the mother nor in the child, and in 28 cases the documentation was missing. Three cases had Down syndrome, one had ring chromosome 18 syndrome, and one had a suspicion of brain-lung-thyroid syndrome.

### 2.4. Maternal TSH and fT4 Values

The documentation of maternal TSH and fT4 values in serum was incomplete. From the group of cases with pCH, 13 out of 72 were missing, and from the group with tCH, 6 out of 20 were missing. The serum TSH and fT4 values of mothers of children with pCH were 1.10 mU/L (0.82–1.40) and 13.40 pmol/L (11.70–16.10), respectively, and from the transient group they were 1.45 mU/L (0.65–2.35) and 14.60 pmol/L (12.35–15.88), for the median and interquartile range, respectively.

### 2.5. Family History

**Figure 4:**
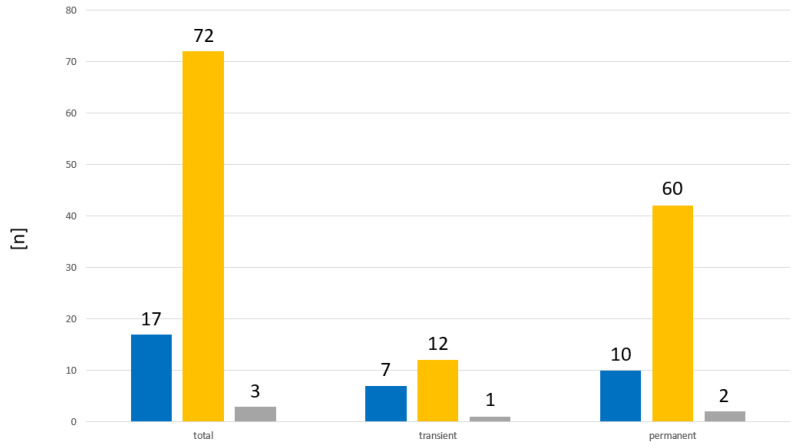
Distribution of family history in patients with CH. ■ Positive family history. ■ Negative family history. ■ Family history not recorded.

From three children, two with permanent and one with tCH, no family history was recorded. Of the remaining 89 cases, 17 (19%) had a positive family history, and in 72 cases (81%), no thyroid disorders were known within first degree relatives (Figure 4). From the remaining 19 cases with tCH, there were 7 cases (37%) with a positive family history. From the remaining 70 cases with pCH, there were 10 cases (14%) with a positive family history.

### 2.6. Whole Blood TSH and TT4 Values of the Neonates

**Figure 5:**
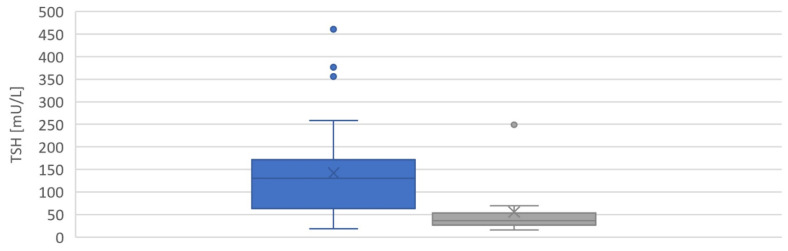
Distribution of TSH values in [mU/L whole blood] from patients with CH at birth. ■ pCH ■ tCH.

**Figure 6:**
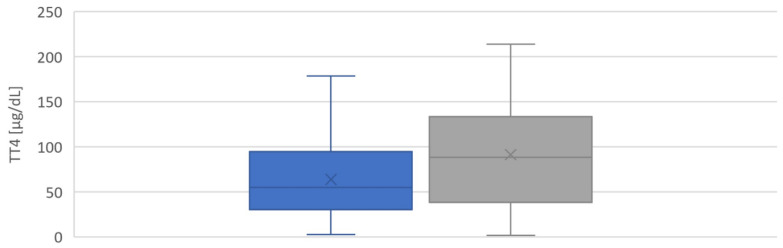
Distribution of TT4 values in [μg/dL whole blood] from patients with CH at birth. ■ pCH ■ tCH.

Whole blood TSH and TT4 values from dried blood spots (DBS) in newborns with pCH were 130.3 (62.9–171.9) mU/L and 55.0 (30.3–94.8) µg/dL (median and interquartile range), respectively. For newborns with tCH, they were 36.4 (26.5–53.3) mU/L and 88.3 (38.6–133.6) µg/dL (median and interquartile range), respectively (Figure 5 and Figure 6). Whole blood TSH values were significantly higher in newborns with pCH (*p* < 0.001) (Figure 5). In the group with tCH, there was one extreme outlier (TSH = 249 mU/L). In addition, values for whole blood TT4 were also lower in newborns with pCH, but the difference was not significant (*p* = 0.1).

**Figure 7:**
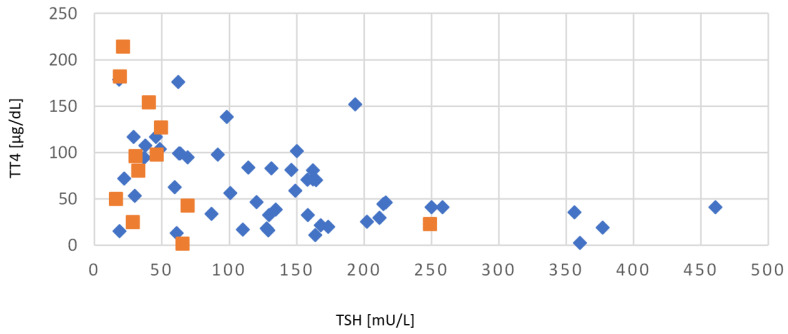
Correlation of whole blood TSH and TT4 values from patients with CH at birth. ♦ pCH ■ tCH.

Figure 7 shows the distribution and correlation of whole blood TSH and TT4 values at birth for children with CH. Whole blood TSH values in the group of tCH were in the range of 18.9–65.3 mU/L, with the additional above mentioned extreme outlier (TSH = 249 mU/L). Cases with pCH showed a much broader variance in whole blood TSH values between 18.1–460 mU. The pearson correlation coefficient (r) showed a significant negative correlation of −0.46, with an r^2^ of 0.22, a t-value of −4.07, and a *p*-value of 0.001.

### 2.7. Sonography of the Thyroid

**Figure 8:**
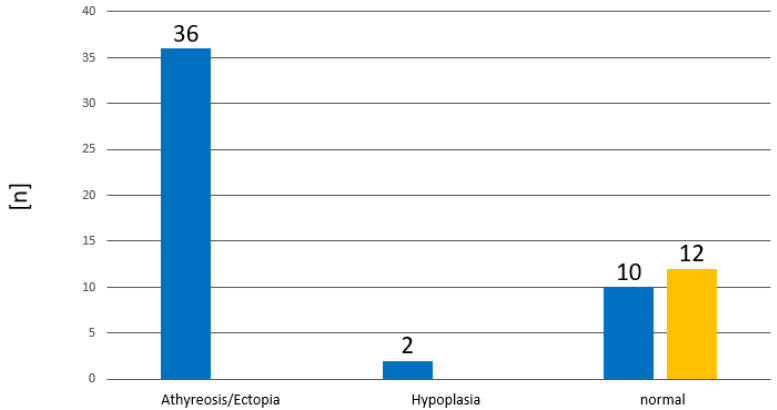
Distribution of diagnostic findings of thyroid sonography. ■ pCH ■ tCH.

**Figure 9:**
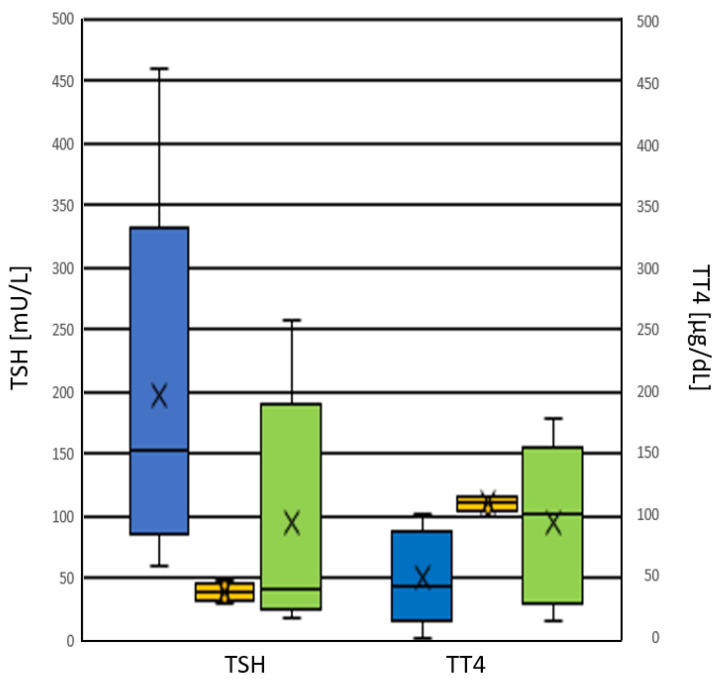
Correlation between thyroid sonography and TSH and TT4, respectively. ■ Athyreosis/ektopia, ■ hypoplasia, ■ normal.

Thyroid sonography is carried out with a linear-array ultrasound transducer with a high resolution of 14 to 18 megahertz. The documentation of thyroid sonography was available in 60 cases (Figure 8; Table 5). In total, 38 had dysgenesis, of which 36 had no thyroid tissue detectable (athyreosis or ectopic thyroid tissue) and two had hypoplasia. All cases with tCH had a normal finding. This means that the positive predictive value of an abnormal thyroid sonography is 100% for pCH. Children with athyreosis or ectopic thyroid tissue had significantly higher TSH and lower TT4 concentrations at birth, compared to the children with hypoplasia (Figure 9). However, it has to be emphasized that there were only two cases with hypoplasia in our cohort.

### 2.8. Gestational Age

In two cases with pCH, there was no documentation of gestational age. Of the remaining 90 cases, 13 were preterm and 75 were term born babies. Of the 13 preterm born babies, 6 had pCH and 7 had tCH. In the group of newborns with pCH 7.8% were preterm, whereas in the group of tCH, 47% were preterm born (Table 6).

The mean GA of the 90 evaluated cases was 38.1 ± 3.6 weeks. The mean GA of the newborns with pCH was 38.7 ± 2.2 weeks, and mean GA of the newborns with tCH was 35.8 ± 6.5 weeks. Babies with tCH showed a lower GA than those with pCH (*p*-value of 0.08, left sided *t*-test).

### 2.9. Birth Weight (bw)

In nine cases with pCH, birth weight was not documented. From the remaining 83 cases, 7 children, all with tCH, had a birth weight < 1500 g. Three had a birth weight < 1000 g, and four between 1000 g–1500 g. In the birth weight group of 1500 g–2500 g, there were five cases with pCH, and one case with tCH (Table 7). The mean birth weight of all 83 cases with CH was 3058 ± 845 g; for the cases with pCH the mean birth weight was 3210 ± 585 g and for the cases with tCH it was 2436 ± 1369 g. Babies with pCH had a significantly higher birth weight than babies with tCH (*p* = 0.04, right sided *t*-test).

### 2.10. Mode of Delivery

The mode of delivery was documented in 47 cases: 35% of newborns with tCH were born by cesarean section, 20% by vaginal delivery, and in 45% of cases documentation was missing. In the group of patients with pCH, 28% were born by cesarean section, 22% by vaginal delivery, and in 50% of cases documentation was missing (Table 8).

## 3. Discussion

In this retrospective cohort study, we found a total incidence of CH of 1:2695, with an incidence of 1:3443 in children with pCH, and of 1:12,396 in children with tCH. Worldwide, the incidence of CH is stated to be 1:2000–1:4000 [7,33], with regional differences and a very high incidence of 1:469 in Turkey [34,35]. Several reasons may explain such a variation. Iodine deficiency can cause tCH [36], and this has been reported for many regions in Turkey [34]. The incidence of tCH seems to have steadily increased during the last years [37]. However, our experience in Switzerland cannot support this notion. We have a very constant incidence of 1:3600 over the whole 24 years of CH screening, despite the fact that the ethnic diversity has changed in Switzerland over the last decades. In addition, the incidence in the canton of Zurich is higher than the mean incidence in Switzerland, but there is so far no explanation for this. There could be several reasons for these discrepant observations: different inclusion and exclusion criteria in the different studies, and a stepwise lowering of the TSH cut-offs without the correct documentation of confirmed diagnosis. In our study, 22% of CH cases had a transient form of CH, which lies within the lower range of formerly published results of 5–65% [10,19,20,21,22]. One possible explanation could be that, in our study, only children that initially needed treatment were defined as having CH. Cases with transient hypertyrotropineamia that resolved without any intervention within the first weeks of life were not included in this study and are also not defined as positive cases in the Swiss NBS Program. In addition, ethnical, environmental, and genetic factors, as well as autoimmunity, were already described as possible factors that influence the incidence of CH [16,38,39,40].

Besides the definite diagnoses of pCH vs. tCH and initial TSH values, additional data from the newborn with CH and from their mothers and families were collected: TT4 at birth, birth weight, gestational age, sex, sonography of the thyroid gland, autoantibodies from the child and the mother, maternal TSH and fT4 values, and family history. Prematurity is primarily defined by GA. We could show that patients with tCH had lower GA and BW. Initial TSH values of the newborns with pCH had significantly higher TSH values than newborns with tCH. Apart from one outlier, all cases with tCH had TSH values <70 mU/L. Therefore, we can draw the reverse conclusion that initial TSH values have a positive predictive value (PPV) of nearly 100%, for pCH. In contrast, TT4 values have no predictive value. In all cases but two, where autoantibodies were detected in the mother, they were also detected in the newborn, however there was no case with positive antibodies in a child, when the antibodies were negative in the mother. The proportion of antibody positive cases was much higher in the group of tCH (67%) compared to the group of pCH (18%). Sonography of the thyroid gland seems to be the best predictor for pCH. Since all cases with tCH had normal findings, an abnormal sonography of the thyroid gland has a 100% PPV for pCH.

The most interesting findings of our study are the relation between the two groups of CH and sex and family history. In the group of tCH, there were equal numbers of cases with a positive and negative family history, and the sex ratio (f/m) was 1:1. In contrast, the group of pCH had a 7-times higher proportion of cases with no family history, and the sex ratio (f/m) was 2.8:1. This finding is in line with results from Quebec [41].

## 4. Materials and Methods

### 4.1. Inclusion and Exclusion Criteria

For our study, we used data from all children that were born between 1 January 2000 and 30 July 2016 and that were finally diagnosed and treated at the University Children’s Hospital Zurich. There were two reasons for this predefinition. First, children had to be at least 2 years old at the start of the study. At 2 years of age, there is a standardized reevaluation of the thyroid function in all children with sonographically detectable thyroid tissue. Thyroxine supplementation was stopped, and then thyroid function was controlled 3–4 weeks after the termination of treatment. In case of normal thyroid function, the latter is controlled in decreasing intervals. Second, patient data had to be easily available, either electronically or by microfilm. Exclusion criteria were missing consent for the use of research data and missing follow-up data at 2 years of age. The reason for the latter could be that the child is deceased or the family has moved out of the canton Zurich. Cases with transient hypertyrotropineamia that resolved without any intervention within the first weeks of life were not included in this study.

### 4.2. Definitions

Newborns with whole blood DBS TSH > 15 mU/L at birth in the NBS sample, which were subsequently diagnosed with CH by means of elevated serum TSH and low or decreased serum fT4 levels, as well as thyroid sonography, were subdivided into pCH and tCH. CH is defined as transient when TSH and fT4 values remain normal after the cessation of thyroxine substitution. So far there are no reference ranges for whole blood TT4 in DBS; however, recently, reference ranges for whole blood TT4 of 83–250 nmol/L (6.4–19.2 µg/dL) in healthy term babies have been published [42]. Reference ranges for newborns and 2-year-old children for serum TSH are 0.7–11 mU/L and 0.7–5.97 mU/L, respectively, and for fT4 they are 11.5–28.3 pmol/L and 12.3–22.8 pmol/L, respectively [43]. Prematurity is defined according to the World Health Organization (WHO) as birth before the end of 37th week of gestation. Sonography of the thyroid was used to distinguish between thyroid dysgenesis, including agenesis, hemiagenesis, ectopy, and normal thyroid development.

### 4.3. Laboratory

All newborns were screened according to the standard protocol of the Swiss NBS Laboratory at the University Children’s Hospital Zurich. No data from the laboratory in Berne were included. Capillary blood is taken by heel prick between 72–96 h of life and dried on special blood collection devices (DBS). TSH and TT4 were measured from 3 mm punches of DBS with the AutoDelfia neonatal test kits from Perkin Elmer, Turku, Finland. TSH, fT4, TPO, and TSHR Ab are measured by standard clinical chemistry methods. In case of elevated TSH, a repeat test in duplicate is performed from the same DBS sample. If TSH remains elevated, TT4 is also measured in the same DBS sample, if the sample provides sufficient blood for its measurement. However, the measurement of TT4 has no influence on the decision to make a recall but decreased TT4 could be informative to speed up a referral. Upon referral, serum TSH and fT4 is always measured. Preterm babies frequently have false positive or false negative results. The reason for false positive results can be the fluctuation of thyroid hormones due to the immaturity of the hypothalamic-pituitary axis, or a reduced conversion of T4 to T3, or a reduced concentration of thyroglobulin. The reason for false negative results is mainly due to a delayed increase of TSH in very preterm babies (<2000 g birth weight; in Switzerland) [44,45]. Therefore, those preterm babies have a second sample taken at 14 days of age.

### 4.4. Sonography of the Thyroid

Sonography of the thyroid is a diagnostic standard in all children with suspicion of CH. In Zurich, it is performed at 3 months of age, after a positive NBS result. A sharp delimitable homogeneous thyroid of median echodensity is defined as normal. In addition, the position of the thyroid is evaluated. A normal pretracheal position can be distinguished from an ectopic positioning along the embryonal ductus thyreoglossus, and from the total absence of the thyroid (athyreosis). The latter is inherently associated with pCH.

### 4.5. Statistics

For the comparison of respective parameters between children with permanent and tCH, we assumed a normal distribution of values. To determine significant differences in TSH values, the right-sided t-test was used, and, for TT4, the left-sided t-test was used. For the evaluation of the significance of the correlation between TSH and TT4 values, the Pearson’s correlation coefficient was calculated at a significance level of 5%. Descriptive statistics are used to delineate family history, sex, and maternal TSH and T4 values. For the determination of a trend in CH, an incidence linear regression analysis and the Von Neumann trend test were performed using the ABACUS 3.0 statistic package from LABanalytics, Jena, Germany.

## 5. Conclusions

Although NBS for CH was already established in the mid-1970s, there are still a lot of unknown details. Until now, a genetic defect has been identified in approx. only 20% of cases [30], however, there is an increasing number of candidate genes for CH (see [46] for commentary). So far, a lot of data from NBS programs are lacking reliable data on confirmatory diagnostics, family history, and long-term follow-up, at least up to 2 years, to confirm permanent or tCH. From the data we present from Switzerland, we could not confirm an increasing incidence of CH, therefore we could also not confirm the hypothesis that the increasing incidence reported from other screening programs could be due to an increased incidence of tCH. To answer this question, screening programs would need to set up long-term follow-up programs to report the final number of cases with tCH and pCH.

## Figures and Tables

**Table 1:** Patient characteristics.

	Permanent CH	Transient CH	Missing Data
Total [n (%)]	72 (78%)	20 (22%)	0
Female [n (%)]	53 (74%)	10 (50%)	0
Male [n (%)]	19(26%)	10 (50%)	0
Birth weight [g]	3210 ± 585	2436 ±1369	1
Gestational age [weeks]	38.7 ± 2.2	35.8 ± 6.5	2
Initial whole blood TSH [mU/L] *	130.3 (62.9–171.9)	36.4 (26.5–53.3)	0
Initial whole blood TT4 [ug/dL] *	55.0 (30.3–94.8)	88.3 (38.6–133.6)	30
Pos. family history [n (%)]	10 (14%)	7 (35%)	3 (3.3%)

* median (interquartile range).

**Table 2:** Incidence of CH in Switzerland from 1977–2021.

Year	Babies Tested	Number of Cases	Incidence	95% CI
1977	73,491	17	1:4323	1:2700–1:7421
1978	72,428	18	1:4024	1:2546–1:6789
1979	72,816	24	1:3034	1:2039–1:4735
1980	75,141	9	1:8349	1:4398–1:18,258
1981	74,539	21	1:3549	1:2322–1:5734
1982	75,940	23	1:3302	1:2200–1:5209
1983	75,284	20	1:3764	1:2437–1:6162
1984	75,426	16	1:4714	1:2903–1:8247
1985	75,852	26	1:2917	1:1991–1:4466
1986	77,898	11	1:7082	1:3958–1:14,186
1987	77,109	20	1:3855	1:2496–1:6312
1988	81,168	28	1:2899	1:2006–1:4363
1989	82,098	25	1:3284	1:1225–1:5074
1990	85,796	26	1:3300	1:2252–1:5025
1991	88,502	23	1:3848	1:2564–1:6070
1992	88,898	23	1:3865	1:2576–1:6097
1993	85,239	28	1:3044	1:2106–1:4581
1994	83,978	24	1:3499	1:2352–1:5461
1995	83,437	24	1:3477	1:2337–1:5426
1996	83,657	29	1:2885	1:2009–1:4307
1997	82,210	30	1:2740	1:1920–1:4062
1998	80,649	13	1:6204	1:3628–1:11,651
1999	79,652	27	1:2950	1:2028–1:4477
2000	79,851	30	1:2662	1:1865–1:3945
2001	74,865	21	1:3565	1:2332–1:5759
2002	74,787	16	1:4674	1:2878–1:8178
2003	74,450	19	1:3918	1:2509–1:6508
2004	75,842	17	1:4461	1:2786–1:7658
2005	76,129	21	1:3625	1:2372–1:5856
2006	76,209	20	1:3810	1:2467–1:6238
2007	77,259	17	1:4545	1:2838–1:7801
2008	79,736	21	1:3797	1:2484–1:6134
2009	81,259	26	1:3125	1:2133–1:4784
2010	83,570	22	1:3799	1:2509–1:6061
2011	83,189	24	1:3466	1:2330–1:5410
2012	86,954	24	1:3623	1:2435–1:5655
2013	85,527	27	1:3168	1:2177–1:4807
2014	86,339	20	1:4317	1:2795–1:7067
2015	88,333	30	1:2944	1:2063–1:4364
2016	88,857	36	1:2468	1:1783–1:3524
2017	88,028	22	1:4001	1:2643–1:6385
2018	88,546	34	1:2604	1:1864–1:3761
2019	85,166	18	1:4731	1:2994–1:7983
2020	88,717	31	1:2862	1:2016–1:4212
2021	91,676	37	1:2478	1:1798–1:3519
1977–2021	3646,497	1038	1:3513	1:3028–1:4180

**Table 3:** Thyroid autoantibodies in the neonatal period in children with tCH and their mothers.

Patient No.		Mother			Child	
	Tg Ab	TPO Ab	TSHR Ab	Tg Ab	TPO Ab	TSHR Ab
1	negative	positive	-	-	positive	negative
2	-	-	-	-	-	-
3	negative	positive	-	-	positive	positive
4	negative	positive	-	positive	positive	
5	negative	negative	-	negative	negative	negative
6	-	-	-	-	-	-
7	-	-	-	-	-	-
8	positive	positive		positive	positive	positive
9	positive	positive	positive	-	-	-
10	-	negative	-	-	negative	-
11–13	-	positive	-	-	positive	-
14	positive	positive	-	positive	positive	-
15	positive	positive	positive	-	positive	-

Tg AB = thyreoglobulin antibodies; TAb = antibodies; TSHR Ab = TSH receptor antibodies.

**Table 4:** Thyroid autoantibodies in the neonatal period in children with pCH and their mothers.

Patient No.		Mother			Child	
	Tg Ab	TPO Ab	TSHR Ab	Tg Ab	TPO Ab	TSHR Ab
1	positive	positive	positive	positive	positive	positive
2	negative	positive	-	positive	positive	positive
3	negative	negative	positive	negative	negative	positive
4	negative	negative	positive	positive	positive	positive
5	positive	positive	negative	negative	negative	-
6	negative	positive	-	positive	positive	negative
7	positive	positive	-	positive	positive	negative
8	negative	positive	negative	negative	positive	negative
9	positive	positive	-	positive	positive	negative
10	negative	negative	positive	negative	negative	negative
11–38	-	-	-	-	-	-
39–72	negative	negative	negative	negative	negative	negative

Tg AB = thyreoglobulin antibodies; TPO Ab = antibodies; TSHR Ab = TSH receptor antibodies.

**Table 5:** Diagnostic findings of thyroid sonography. [n (%)].

	Permanent CH	Transient CH	Total
Dysgenesis	38 (79%)	0 (0%)	38 (63%)
Athyreosis/Ektopia	36 (75%)	0 (0%)	36 (60%)
Hypoplasia	2 (4%)	0 (0%)	2 (3.3%)
Normal	10 (21%)	12 (100%)	22 (36.7%)
Total	48 (100%)	12 (100%)	60 (100%)

**Table 6:** Distribution of term and preterm babies with CH. [n (%)].

	Permanent CH	Transient CH
Preterm babies (GA < 37 weeks)	6 (7.8%)	7 (47%)
Term babies (GA > 37 weeks)	69 (89.6%)	8 (53%)
No documentation	2 (2.6%)	0 (0%)
Total	77 (100%)	15 (100%)

**Table 7:** Distribution of birth weight in babies with CH. [n (%)]. bw: birth weight.

	Permanent CH	Transient CH
bw < 1000 g	0 (0%)	3 (15%)
bw < 1500 g	0 (0%)	4 (20%)
bw > 1500 g and <2500 g	5 (6.9%)	1 (5%)
bw > 2500	58 (80.6%)	12 (60%)
No documentation	9 (12.5)	0 (0%)
Total	72 (100%)	20 (100%)

**Table 8:** Evaluation of delivery mode in babies with CH. [n (%)].

	Permanent CH	Transient CH
Caesarean section	20 (28%)	7 (35%)
Vaginal delivery	16 (22%)	4 (20%)
No documentation	36 (50%)	9 (45%)
Total	72 (100%)	20 (100%)

## Data Availability

Not applicable.
